# Phosphate Limitation Triggers the Dissolution of Precipitated Iron by the Marine Bacterium *Pseudovibrio* sp. FO-BEG1

**DOI:** 10.3389/fmicb.2017.00364

**Published:** 2017-03-14

**Authors:** Stefano Romano, Vladimir Bondarev, Martin Kölling, Thorsten Dittmar, Heide N. Schulz-Vogt

**Affiliations:** ^1^Max Planck Institute for Marine Microbiology,Bremen, Germany; ^2^Marum Center for Marine Environmental Sciences,Bremen, Germany; ^3^ICBM-MPI Bridging Group for Marine Geochemistry, Institute for Chemistry and Biology of the Marine Environment, Carl von Ossietzky University,Oldenburg, Germany; ^4^Biological Oceanography, Leibniz Institut für Ostseeforschung,Warnemünde, Germany

**Keywords:** *Pseudovibrio*, phosphate limitation, siderophores, *Roseobacter*, chelators, metabolomics, bacterial physiology, mass spectrometry

## Abstract

Phosphorus is an essential nutrient for all living organisms. In bacteria, the preferential phosphorus source is phosphate, which is often a limiting macronutrient in many areas of the ocean. The geochemical cycle of phosphorus is strongly interconnected with the cycles of other elements and especially iron, because phosphate tends to adsorb onto iron minerals, such as iron oxide formed in oxic marine environments. Although the response to either iron or phosphate limitation has been investigated in several bacterial species, the metabolic interplay between these two nutrients has rarely been considered. In this study we evaluated the impact of phosphate limitation on the iron metabolism of the marine bacterium *Pseudovibrio* sp. FO-BEG1. We observed that phosphate limitation led to an initial decrease of soluble iron in the culture up to three times higher than under phosphate surplus conditions. Similarly, a decrease in soluble cobalt was more pronounced under phosphate limitation. These data point toward physiological changes induced by phosphate limitation that affect either the cellular surface and therefore the metal adsorption onto it or the cellular metal uptake. We discovered that under phosphate limitation strain FO-BEG1, as well as selected strains of the *Roseobacter* clade, secreted iron-chelating molecules. This leads to the hypothesis that these bacteria might release such molecules to dissolve iron minerals, such as iron-oxyhydroxide, in order to access the adsorbed phosphate. As the adsorption of phosphate onto iron minerals can significantly decrease phosphate concentrations in the environment, the observed release of iron-chelators might represent an as yet unrecognized link between the biogeochemical cycle of phosphorus and iron, and it suggests another biological function of iron-chelating molecules in addition to metal-scavenging.

## Introduction

Phosphorus (P) is an important component of biomolecules and a fundamental element in cellular regulatory processes. Bacteria acquire phosphorus via phosphate (P_i_), which in natural environments is often scarce. In marine waters, the concentration of P_i_ may be in the nanomolar range, thus limiting bacterial growth and productivity in several areas of the ocean, at least during part of the year (Cotner et al., 1997; Wu et al., 2000; Thingstad et al., 2005). Bacteria evolved a sophisticated system to overcome suboptimal P availability, and it has been shown that P_i_ limitation affects not only cellular P metabolism, but also central metabolism, production of secondary metabolites, and the expression of virulence-related genes (Martín, 2004; Hsieh and Wanner, 2010). But while this response has been well-studied in model organisms and organisms of biotechnological interest, such as *Escherichia coli*, *Sinorhizobium meliloti*, and *Streptomyces coelicolor*, less is known about the response of heterotrophic marine bacteria (Ishige et al., 2003; Krol and Becker, 2004; Rodríguez-García et al., 2007; Romano et al., 2015).

In the ocean, the geochemical P cycle is tightly linked to the geochemical cycles of other elements, especially iron (Fe), given that P_i_ strongly adsorbs onto Fe minerals. Indeed, in some parts of the ocean this process removes up to 40% of the total soluble P_i_ input (Berner, 1973; Froelich et al., 1977; Feely et al., 1998; Châtellier et al., 2013). This phenomenon has been also described in freshwater systems (Cosmidis et al., 2014). Like P, Fe is often a limiting micronutrient in marine surface waters. Under oxic conditions Fe(III) is the only thermodynamically stable form, but its solubility at circumneutral pH is several orders of magnitude lower than the average amount of Fe required for bacterial growth (Millero, 1998; Andrews et al., 2003). Due to its low solubility and concentration in oxic environments, microorganisms produce and secrete organic molecules, siderophores, that bind Fe(III) with high affinity and are able to dissolve Fe particles, thus facilitating Fe acquisition. These molecules are produced by microorganisms in response to Fe limitation, and allow them to scavenge Fe from the environment (Wandersman and Delepelaire, 2004).

Biogeochemical studies have linked the concentration of P_i_ with the concentrations of other micronutrients in different areas of the ocean (Saito and Moffett, 2002; Paytan and McLaughlin, 2007; Jakuba et al., 2008). Moreover, molecular studies have underlined how different enzymes involved in P_i_ and organo-P_i_ metabolism have metal ions as cofactor (Kulakova et al., 2003; Sebastian and Ammerman, 2009; Kathuria and Martiny, 2011; Yong et al., 2014). All together these data suggest that the metabolism of P_i_ is closely coupled to that of other micronutrients in microorganisms. However, little is known about the effect of P_i_ limitation on the metabolism of Fe and other micronutrients in bacteria, and, to the best of our knowledge there have been no such studies in marine heterotrophic bacteria. In previous proteomic and metabolomic studies we showed that the activation of the P_i_ starvation response, as a consequence of P_i_-limited growth, has a drastic impact on both the central and the secondary metabolism of strain FO-BEG1 (Romano et al., 2014, 2015), a marine heterotrophic bacterium belonging to the broadly distributed and metabolically versatile genus *Pseudovibrio* (Bondarev et al., 2013; Schwedt et al., 2015; Romano et al., 2016). In this study we aimed to investigate the impact of P_i_ limitation on the micronutrient metabolism, especially that of Fe, of strain FO-BEG1. Surprisingly, we observed that P_i_ limitation triggered the secretion of Fe-chelating molecules into the medium, a phenomenon which was also observed to a certain extent in cultures of strains belonging to the abundant and ubiquitous *Roseobacter* clade.

## Materials and Methods

### Growth Conditions

Strain FO-BEG1 was cultivated under P_i_-limited (−P_i_; C:N:P ratio: 600:100:1) and P_i_-surplus conditions (+P_i_; C:N:P ratio: 43:7:1) in carbohydrate mineral medium (CMM), containing per liter: 15 g NaCl; 1.5 g MgCl_2_ hexahydrate; 1 g K_2_SO_4_; 0.27 g (10 mmol L^−1^) NH_4_Cl; 0.005 g CaCl_2_ (Bondarev et al., 2013; Romano et al., 2015). The medium was buffered using Tris-HCl (3.9 g L^−1^), brought to pH 8, and after autoclaving Fe(II), and Co were supplied to a final concentration of 7.5 and 0.75 μmol L^−1^, respectively, together with other micronutrients from an acidified trace-element solution (Widdel and Pfennig, 1984). Additionally, the medium was amended with glucose (10 mmol L^−1^), 1 ml L^−1^ tungsten/selenium solution (Brysch et al., 1987), 1 ml L^−1^ of four vitamin solutions (prepared according to Aeckersberg et al., 1991), and for the P_i_ surplus conditions, P_i_ was added from a K_2_HPO_4_ solution, to a final concentration of 1.4 mmol L^−1^. In the P_i_-limited medium no P_i_ was added, and the only P_i_ source (100 μmol L^−1^) derived from the buffer used to prepare the vitamin solutions. The cultures were incubated at 28°C in the dark under shaking conditions (120 rpm). All *Roseobacter* strains, were purchased from the German Collection of Microorganisms and Cell Cultures (DSMZ), and were similarly cultivated and incubated at 28°C (*Phaeobacter inhibens* DSM-17395, DSM-16374) or 30°C (*Ruegeria pomeroyi* DSM-15171). All experiments were performed using a 0.001% v/v inoculum, consisting of cells grown under +P_i_ conditions. Bacterial growth was monitored by measuring the optical density (OD) at 600 nm using an Eppendorf BioPhotometer (Eppendorf AG, Germany). The OD_600_ was then correlated with the cell number, determined using a counting chamber (Brand GmbH, Germany; data not shown). All cultivation experiments were performed in biological triplicates and included controls, consisting of sterile un-inoculated media, which were incubated under the same conditions described for the cultures. For all cultures and sterile controls, the Fe and Co concentrations in the cell-free supernatants were determined over the course of the incubations using inductively coupled plasma optical emission spectroscopy (ICP-OES), and P_i_ was determined colorimetrically using the ammonium molybdate method (Hansen and Karoleff, 1999).

To assess whether the increase in the Fe concentration measured during P_i_-limited growth of strain FO-BEG1 was due to dissolution of the precipitated Fe fraction, two experiments, hereafter referred to as the “refresh experiment” and the “EDTA experiment,” were performed. A schematic overview of the cultivation approaches used for strain FO-BEG1 is reported in **Supplementary Figure S1**. In the refresh experiment (**Supplementary Figure S1B**), strain FO-BEG1 was incubated in CMM under −P_i_ conditions until Fe uptake reached the previously detected maximum. The biomass of all replicates was then harvested by centrifugation at 7,000 ×*g* and 15°C for 10 min using a J-26XP Beckmann centrifuge and a JA-10 rotor (Beckman Instruments, Inc., Palo Alto, CA, USA). The resulting cell pellets were washed with Fe-free sterile artificial seawater and then equally divided to re-inoculate fresh Fe-free P_i_-limited CMM in biological triplicates. These cultures were incubated again in the dark at 28°C with shaking at 120 rpm. Bacterial growth was then monitored for 95 h by measuring the OD_600_. The Fe concentration in the cell-free supernatants was determined by ICP-OES. For this second cultivation step, residual Fe adsorbed to the glass was removed by washing twice, prior to use, all glassware with 1 M HCl followed by rinsing with MembraPure water (Optilab-Standard Water System, MembraPure, Bodenheim, Germany).

In the EDTA experiment (**Supplementary Figure S1C**), strain FO-BEG1 was cultivated in +P_i_ and −P_i_ CMM, both prepared using an acidified trace-element solution not containing Fe, which was instead supplied separately as EDTA-complex to a final concentration of 7.5 μmol Fe L^−1^ and 100 μmol EDTA L^−1^. The cultures were incubated as described above and all glassware was acid-washed prior to use. The Fe concentrations in the cell-free supernatants were determined by ICP-OES. Fe-chelating molecules were extracted from the cell-free supernatant of a 10 L culture grown in the −P_i_ CMM, supplemented with the original acidified trace element solution, in a Sartorius bioreactor with a Biostat B Plus control unit (Sartorious GmbH, Göttingen, Germany). The temperature was maintained at 28°C and the culture was mixed at 300 rpm. Filtered air was injected into the bioreactor at a constant flow rate of 1 L min^−1^. Throughout the incubation time, both pH and bacterial growth (OD_600__nm_) were monitored.

### Trace Metal Measurement, Dialysis, and Size-Exclusion Chromatography (SEC) of the Cell-Free Supernatants

The Fe and Co concentrations in the supernatants were measured using ICP-OES. The samples were collected during bacterial growth, centrifuged at 11,000 ×*g* and 5°C for 10 min, filtered through Millipore filters (0.22-μm pore size; Millipore, Bedford, MA, USA), diluted 1:10 in 1% supra-pure HNO_3_ (Merck, Darmstadt, Germany), and stored at 4°C until further processing. They were analyzed using an Agilent 720 ICP-OES (Agilent Technologies, Palo Alto, CA, USA). Cell-free supernatants of the −P_i_ cultures were dialyzed overnight at 4°C against MQ under stirring conditions using a Spectra/Por TM dialysis tube (molecular cut-off: 1000 Da; Serva, Heidelberg, Germany). The Fe content was measured before and after dialysis via ICP-OES.

Size-exclusion chromatography (SEC) of the −P_i_ cell-free supernatant was performed by injecting 1 mL of sample into an Äkta purifier system (GE Healthcare, Freiburg, Germany) equipped with a Superdex^TM^ peptide 10/300 GL column (GE Healthcare, Freiburg, Germany), detecting absorption at 280 nm. The samples were run using the buffer Tris(hydroxymethyl)aminomethane hydrochloride (Tris-HCl), at pH 7.8 and a flow rate of 0.25 mL min^−1^. During the chromatographic run, 1 mL fractions were collected using a Frac-950 fraction collector (GE Healthcare, Piscataway, NJ, USA). The column was calibrated using ribonuclease A, aprotinin, [d-Ala^2^, d-Leu^5^]-enkephalin, and vitamin B12 (Sigma-Aldrich Chemie Gmbh, Munich, Germany), all of which were run under the above-described conditions. The total volume of the column was estimated based on the elution time of the ions in solution, detected as an increase in conductivity. Each fraction was then diluted 1:10 in 1% supra-pure HNO_3_ and its Fe content was analyzed using ICP-OES.

### Assay of the Chelating Activity and Partial Purification of the Chelating Compounds

The presence of Fe-chelating molecules in the cell-free supernatants of cultures grown in CMM, supplemented with the original acidified trace element solution, was tested using the chromazurol-S (CAS) assay, modified from Schwyn and Neilands (1987). To 0.6 mL of 10 mmol L^−1^ hexadecyltrimethylammonium bromide (HDTMA), 0.15 mL of 1 mmol L^−1^ FeCl_3_, prepared in 0.1 mol L^−1^ HCl, was slowly added under stirring, followed by the dropwise addition of 0.75 mL of 2 mmol L^−1^ CAS. After complete mixing of the solution, 6.5 mL of 0.5 mmol L^−1^ piperazine-*N,N*′-bis(2-ethanesulfonic acid) (PIPES), previously adjusted to pH 6.8, was added. MembraPure water was then added to achieve a final volume of 10 mL. The CAS solution was always prepared fresh before use, and the assay was performed using a mixture of equal volumes of CAS solution and sample.

A crude extract was prepared from a 10 L cell-free supernatant as follows. The cells from a 10 L culture were harvested by centrifugation at 11,000 ×*g* and 5°C for 20 min using a J-26XP Beckmann centrifuge and a JA-10 rotor (Beckman Instruments) and filtered through a 0.22-μm membrane filter (Sartorius AG, Göttingen, Germany). The cell-free supernatant was then acidified to pH 2.6 using 5 mol HCl L^−1^ and mixed in the dark for 1 h with an equal volume of ethyl acetate. The solvent phase was collected and the solvent was evaporated in a Laborota 4,000 rotary evaporation system (Heidolph, Schwabach, Germany) at 45°C. Evaporation was completed in a speed-vacuum centrifuge (Eppendorf Concentrator 5301, Eppendorf, Hamburg, Germany). Prior to isolating the compounds using high performance liquid chromatography (HPLC), the activity of the crude extract was confirmed using the CAS assay.

The chelating molecules were isolated in collaboration with BioViotica GmbH (Göttingen, Germany), using the extract obtained from the above mentioned 10 L culture. The complexity of the crude extract was verified in an analytical reverse-phase HPLC run using a C18 column (Nucleodur 100-5 C18ec, 250 mm × 3 mm). Eluents A and B were 100% MQ and 70% MQ and 30% acetonitrile (ACN), respectively, both containing 0.1% trifluoroacetic acid, which was handled under chemical safety conditions. The program for the separation was: 0–20 min, from 20% B to 100% B, 20–30 min 100% B, and 30–35 min, from 100% B to 20% B, at a flow rate of 2.5 mL min^−1^. The same conditions were then applied to isolate the metabolites using a preparative C18 column (Kromasil 100-7 C18, 250 mm × 20 mm) at a flow rate of 18 mL min^−1^ and recording the absorption at 300 nm. The fractions were manually collected during the preparative HPLC runs every time an increase in the absorption was observed. The eluent was evaporated and the fractions were re-dissolved in 70% ACN to test their activity in the CAS assay. The composition of the active fraction 1 was verified by performing two more analytical runs, using two C18 analytical columns (Nucleodur 100-5 C18ec, 250 mm × 3 mm; Phenomenex aqua C18, 250 mm × 2.0 mm) and the above-described running conditions.

### Electrospray Ionization Fourier Transform Ion Cyclotron Resonance Mass Spectrometry (ESI-FT-ICR-MS) of the Reverse-Phase HPLC Fractions

The HPLC fractions resulted positive in the CAS-assay, fractions 1 and 4, were further analyzed via mass spectrometry (MS). The fractions were diluted 1:10 in 70% ACN and filtered through a PTFE filter (0.2-μm pore size; Rotilabo, Carl Roth GmbH, Karlsruhe, Germany). MS analysis was performed in electrospray ionization (ESI) positive mode with a solariX FT-ICR-MS (Bruker Daltonik GmbH, Bremen, Germany) equipped with a 15.0 Tesla magnet. All data were acquired with a time domain size of 4 megawords with a detection range of *m/z* 150–2,500. For each run, 200 broadband scans were accumulated. To facilitate the identification of chelating molecules both HPLC fractions were analyzed before and after the addition of analytical grade GaCl_3_ (Sigma-Aldrich Chemie GmbH, Munich, Germany). The acquired mass spectra were analyzed using the Data Analysis software v4.0 SP4 (Bruker Daltonik GmbH, Bremen, Germany). An external mass calibration was performed for all spectra, which were then manually inspected to identify masses showing the characteristic Ga isotopic signature. Since, the two Ga isotopes, ^69^Ga and ^71^Ga, have an abundance ratio of 3:2, Ga-containing compounds can be unambiguously identified considering the mass differences and the ratio of the signal intensities of the isotopologues.

### Bioinformatics Analysis

The *in silico* prediction of gene clusters involved in the biosynthesis of siderophores was performed by analyzing both genomic and plasmid sequences of *Pseudovibrio* sp. FO-BEG1 (GenBank ID: CP003147.1 and CP003148.1, respectively) with antiSMASH v3.0 (Weber et al., 2015). The same analysis was performed for the genomes and plasmids of *P. inhibens* strain DSM-17395 (GenBank ID: CP002976.1, CP002977.1, CP002979.1, CP002978.1) and of *R. pomeroyi* (CP000031.1, CP000032.1). The assembly of *P. inhibens* strain DSM-16374 (GenBank ID: GCA_000154765.2) was annotated using PROKKA v1.10 (Seemann, 2014) and then submitted to antiSMASH as well.

## Results

### Micronutrient Measurements

**FIGURE 1:**
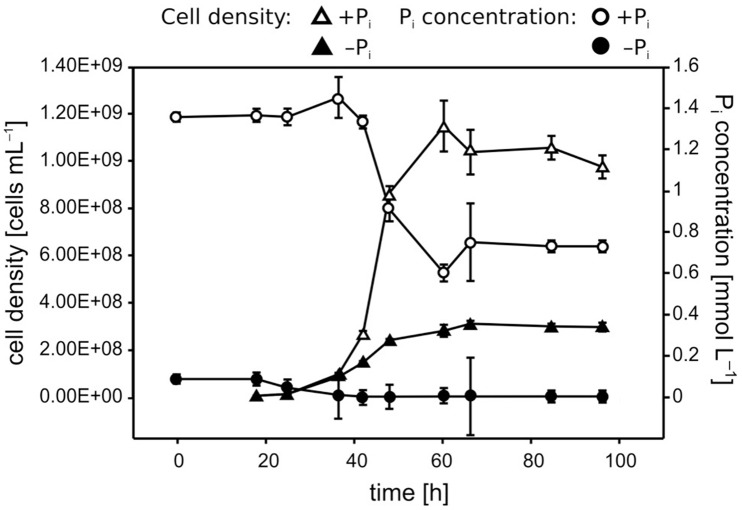
**Growth and P_i_ uptake of *Pseudivbrio* sp. strain FO-BEG1 under the two P_i_ regimes.** P_i_-limited and P_i_-surplus conditions are indicated with filled and empty symbols, respectively. Triangles indicate the cell densities and circles indicate the P_i_ concentrations over time. Error bars represent the standard deviations of the data as determined from biological triplicates. The data refer to a different experiment than the one described in **Figure 2**.

**FIGURE 2:**
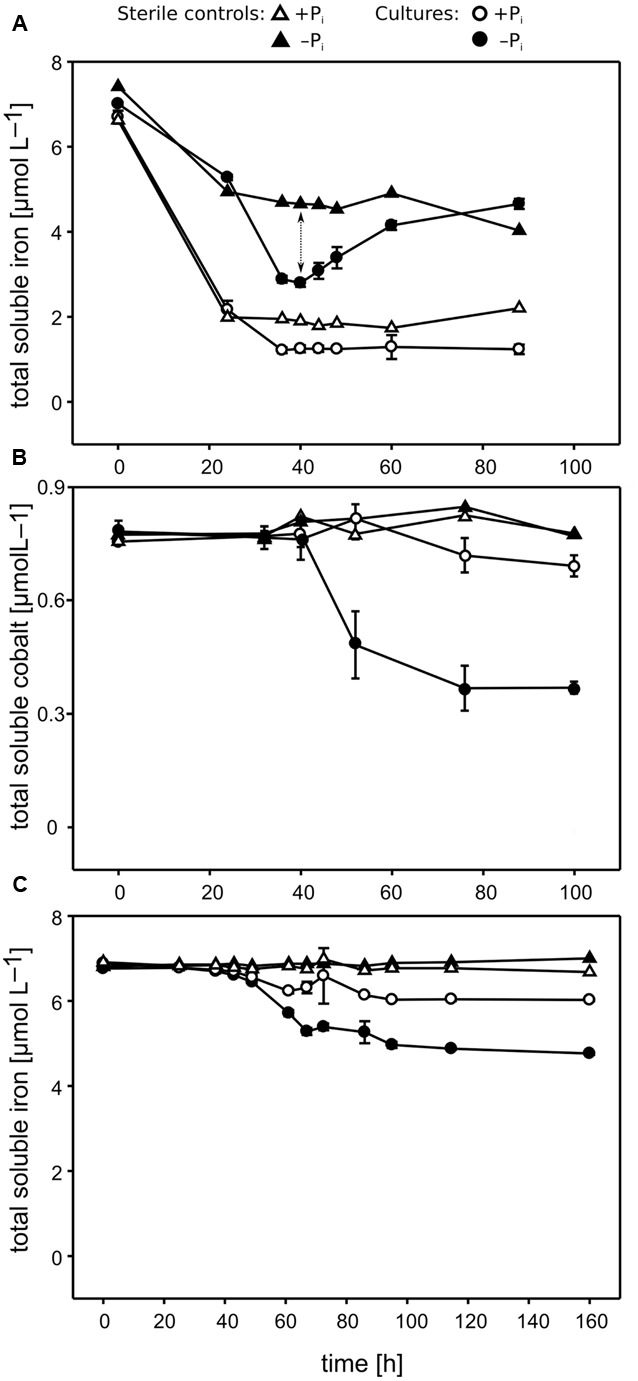
**Concentrations of micronutrients over time measured in *Pseudivbrio* sp. strain FO-BEG1 cultures. (A,B)** Show the Fe and Co concentrations, respectively, in the original cultivation experiments. **(C)** Shows the Fe concentrations in the EDTA experiment. Filled symbols indicate −P_i_ growing cultures, and open symbols cultures growing under +P_i_ conditions. Total biotic Fe removal (indicated with a dotted arrow in **A**) can be inferred from the difference between the Fe concentrations in the sterile controls (triangles) and the cultures (circles). Error bars represent the standard deviations based on biological triplicates. The data refer to a different experiment than the one described in **Figure 1**.

**FIGURE 3:**
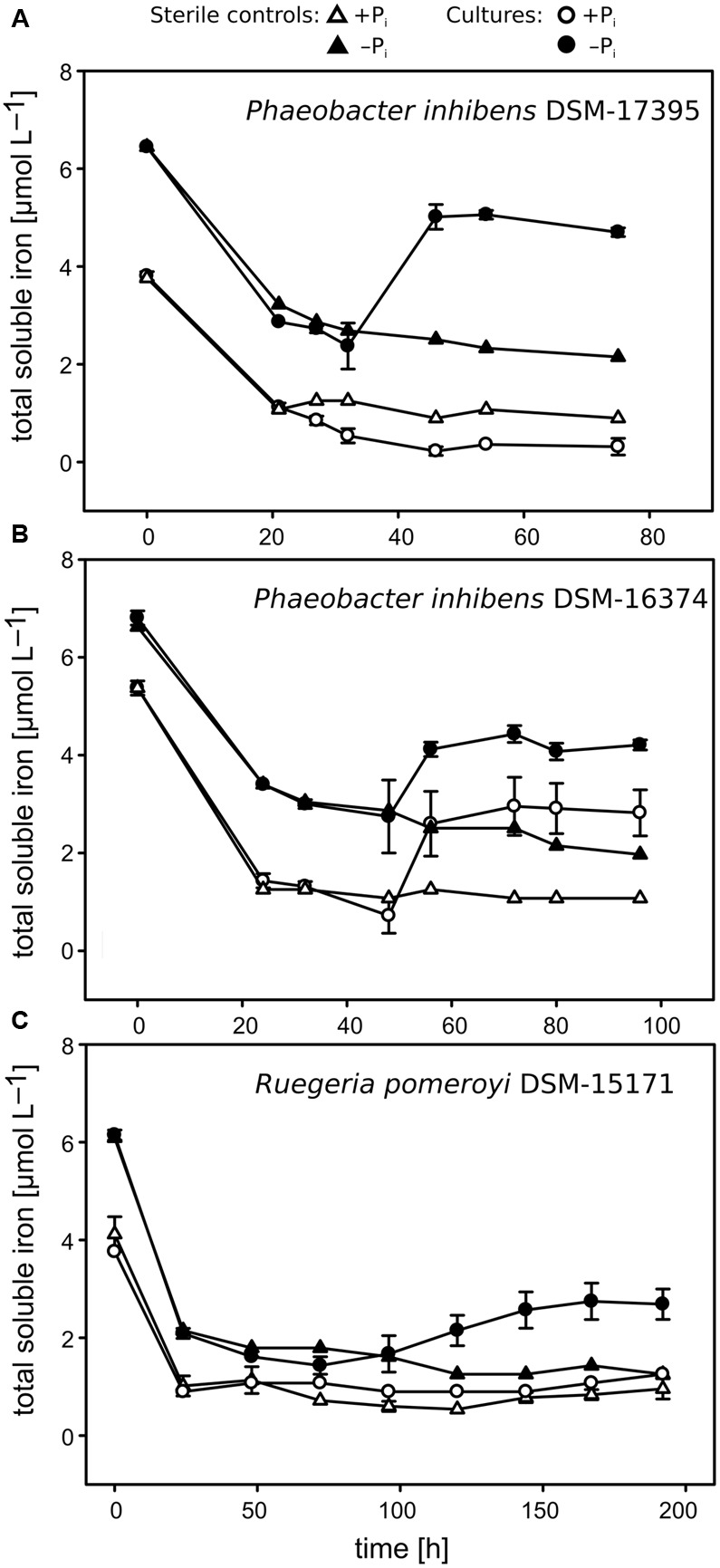
**Concentrations of total soluble Fe over time measured in the cultures of the *Roseobacter* strains.** Sterile controls are indicated with triangles, and cell-free supernatants of the cultures are indicated with circles. **(A)** *Phaeobacter inhibens* strain DSM-17395; **(B)** *P. inhibens* strain DSM-16374; **(C)** *Ruegeria pomeroyi* strain DSM-15171. Filled symbols represent cultures growing under −P_i_ conditions and open symbols cultures growing under +P_i_ conditions. Error bars represent the standard deviations based on biological triplicates. Different initial Fe concentrations between the two P_i_ regimes were likely due to the differential precipitation rates of Fe under low and high P_i_ amounts occurred during the period of time needed for sample preparation.

P_i_ limitation repressed *Pseudovibrio* growth, leading to a final cell density 2.5- to 3.5-fold lower than observed under +P_i_ conditions (**Figure 1**). Cells growing under P_i_ surplus did not metabolize all of the P_i_ provided, whereas P_i_ was taken up completely under −P_i_ conditions during the first ∼30 h of growth, reaching our detection limit of ∼3 μmol L^−1^ (**Figure 1**). In the sterile controls abiotic Fe precipitation occurred in both the +P_i_ and −P_i_ media, but it was more pronounced under +P_i_ conditions (**Figure 2A**). Fe was added to the medium as Fe(II), which oxidized to Fe(III) during the oxic incubation. The solubility of Fe(III) in natural seawater at circumneutral pH is below 1 nmol L^−1^ (Millero, 1998), which explains the strong precipitation in the CMM. The presence of residual Fe in solution might be explained by the presence of the Tris buffer, which can interact with metal ions (Morrow et al., 1992). Considering that the only difference in medium composition between the two treatments was the P_i_ concentration, it is most likely that the higher P_i_ concentrations, or higher concentrations of its counter cations K, were responsible for the increased Fe precipitation. In the cultures both abiotic and biotic processes influenced the Fe concentrations. The biotic processes include cellular uptake and adsorption onto cellular surface, and can be estimated by calculating the difference between the minimum Fe concentrations measured in the cultures and the sterile controls. The biotic Fe removal was 1.5- to 3-fold higher under P_i_ limitation than under P_i_ surplus conditions (**Figure 2A**). Surprisingly, also the biotic Co removal from the media showed significant differences between the two P_i_ regimes. Under P_i_-limited conditions biotic Co removal was 3- to 5-fold higher than that observed under P_i_ surplus (**Figure 2B**). In contrast to this, in none of the cultures of the *Roseobacter* strains biotic Fe and Co removal differed between the two P_i_ regimes (**Figure 3**, data not shown for Co).

### Total Soluble Iron Increases during Bacterial Growth under P_i_ Limitation

Following the Fe concentration in **Figure 2A**, it can be seen that only under −P_i_ conditions the concentration of the total soluble Fe increased again during the late bacterial growth. This phenomenon always started in the late exponential phase and continued during the stationary phase. Theoretically, the increase in Fe may have derived from either the cellular release or the dissolution of the precipitated fraction. We hypothesized that strain FO-BEG1 released molecules able to dissolve the Fe that abiotically precipitated in the medium. In order to test this hypothesis we performed two experiments, hereafter referred to as the “refresh experiment” and the “EDTA experiment” (**Supplementary Figure S1**). In the refresh experiment, cells were pre-grown under −P_i_ conditions, until the previously detected maximum Fe uptake was reached (**Figure 2A**). Cells were then harvested and re-inoculated into fresh Fe-free −P_i_ CMM. In this experiment, we hypothesized that since the fresh medium did not contain Fe, any Fe increase would have indicated cellular release. In the EDTA-experiment, strain FO-BEG1 was cultivated in +P_i_ and −P_i_ CMM containing an excess of EDTA, in order to complex Fe. Consequently, Fe precipitation would not occur neither in the sterile controls nor in the cultures and a release of Fe-chelators by the cells would not result in an increase of total soluble Fe in the cultures. On the other hand, a release of stored Fe by the cells would increase the total soluble Fe in the cultures, as this Fe would be kept in solution by the excess EDTA.

The results of both experiments clarified that the Fe increase, observed in the original cultivation experiments (**Supplementary Figures S1A, 2A**), derived from the dissolution of the precipitated fraction, because we did not observe any Fe increase in both the refresh experiment (data not shown) and the EDTA experiment (**Figure 2C**). As hypothesized, in the EDTA experiment, the addition of EDTA prevented Fe precipitation (see the data for the sterile controls in **Figure 2C**) and, consistent with the original cultivation approach (**Figure 2A**), P_i_ limitation induced a ∼2.5-fold higher biotic Fe removal. Also, the EDTA experiment showed that the biotic Fe removal did not continue throughout the late phase of growth, when Fe increased in the first cultivation experiments performed using a CMM amended with uncomplexed Fe(II) (**Figures 2A,C** and **Supplementary Figures S1A,C**). This suggests that the bacteria did not continuously took up Fe during the late growth phase. In order to understand whether the secretion of Fe-chelators under P_i_ limitation is a general phenomenon observed amongst heterotrophic marine bacteria, we performed similar analyses in three strains of the abundant and ubiquitous *Roseobacter* clade: *P. inhibens* DSM-17395, DSM-16374 and *R. pomeroyi* DSM-15171. As in strain FO-BEG1, in strains DSM-17395 and DSM-15171 the soluble Fe increased during the late growth phase only under −P_i_ conditions (**Figures 3A,C** and **Supplementary Figure S2**) whereas in strain DSM-16374 Fe increased in both the −P_i_ and +P_i_ cultures (**Figure 3B**). It is worth noting that strain DSM-16374 increasingly formed tight flocs throughout growth, creating a bias in the cell density estimation (**Supplementary Figure S2B**).

### Detection of Chelating Molecules in the Cell-Free Supernatant of P_i_-Limited *Pseudovibrio* Cultures

**FIGURE 4:**
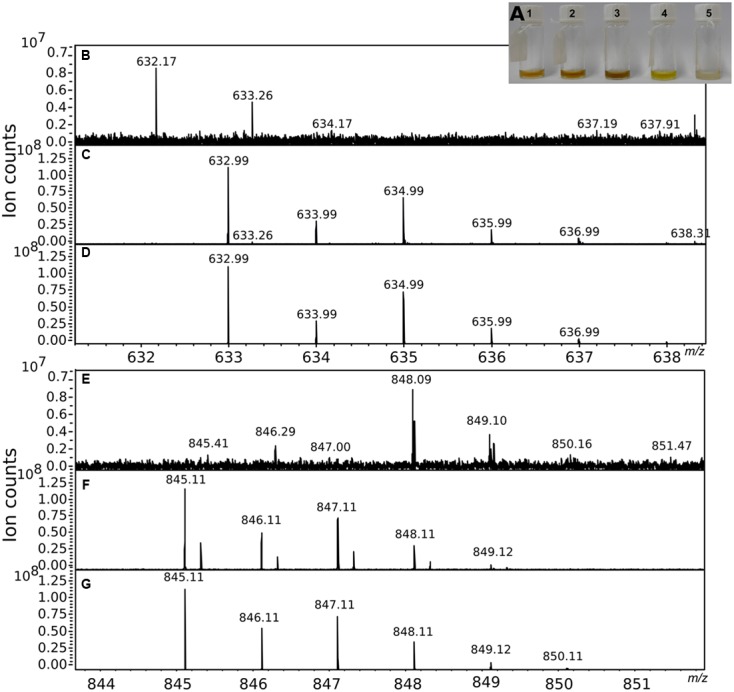
**Partial purification of the chelating agents. (A)** Shows the five fractions obtained from the preparative HPLC fractionation performed using the crude extract obtained from a 10 L −P_i_ culture. The chelating activity of the fractions was tested in the CAS assay and confirmed by mass spectrometry. **(B,E)** Show a portion of the mass spectra of fraction 1. **(C,F)** Show the mass spectra of the same portions after GaCl_3_ addition. The characteristic isotopic pattern created by the Ga isotopes was compared to a theoretical pattern **(D,G)** calculated for a molecule of the same mass, allowing for the presence of a maximum of one atom of Ga in the final molecular formula.

The secretion of Fe-chelating molecules by *Pseudovibrio* under Pi limitation was then confirmed via chromatographic and mass spectrometric techniques. Fractionation of the −P_i_ supernatant by SEC followed by measurement of the Fe content of each fraction using ICP-OES showed that Fe was indeed bound to organic molecules present in solution in the cell-free supernatants. The apparent molecular sizes of those compounds were between 1,030 and 4,885 Da. These data were consistent with the dialysis experiment performed using the −P_i_ cell free supernatant and a membrane with a molecular cutoff of 1 kDa. During the dialysis process, the Fe concentration should have decreased if Fe complexes < 1 kDa in size had formed, but we could not observe such a decrease (data not shown). In agreement with these results, in the CAS assay chelating molecules were detected only in the −P_i_ supernatant, a finding confirmed by ultra-high resolution mass spectrometry (FT-ICR-MS). The compounds in the crude extract of the supernatant obtained from a 10 L −P_i_ culture, were separated using HPLC into five fractions (**Figure 4A**). Amongst them, fraction 4 contained a compound with a retention time and UV-visible spectrum consistent with tropodithietic acid (TDA), previously reported to be produced by strain FO-BEG1 under −P_i_ conditions (Romano et al., 2014, 2015). Chelating properties of all fractions were tested again via the CAS-assay. Fraction 1 reacted quickly in the assay, but successive HPLC analyses showed the presence of multiple compounds, which hindered a complete characterization of the chelating agents enriched in this sample. Additionally, the TDA-containing fraction started to react after ∼2 h of incubation.

Fractions 1 and 4 were then analyzed by MS before and after GaCl_3_ addition. Ga(III) has chemical properties similar to Fe(III), but its two isotopes, ^69^Ga and ^71^Ga, have an abundance ratio of 3:2 that facilitates the identification of chelating compounds (Gledhill et al., 2004). In fraction 1 we identified the molecular masses of several compounds (i.e., nominal masses 633; 845; 874), that according to the analysis of the isotopic patterns unequivocally contained Ga (**Figures 4B–G**). An additional peak with a strong intensity was identified at 311 *m/z*. However, a similar *m/z* with an intensity ∼20-fold lower was also detected in a control run performed by dissolving GaCl_3_ in ACN. In the TDA fraction, masses showing the Ga signature were not identified. Instead a peak with a mass (*m/z* 234.9; [TDA+Na]^+^) and isotopic pattern consistent with TDA was detected.

## Discussion

### *Pseudovibrio* Secretes Unknown Fe-Chelating Molecules under Phosphate Limitation

P_i_ and Fe are both fundamental nutrients for all organisms. The interconnection between their geochemical cycles suggests that also their cellular metabolisms are tightly linked; however, there is a dearth of studies considering these metabolic connections in bacteria. By combining physiological and chemical experiments, we report in this work the impact of P_i_ limitation on the Fe metabolism of the marine bacterium *Pseudovibrio* sp. FO-BEG1. The results of the growth experiments together with the chromatographic and mass spectrometry data clearly showed that under P_i_ limitation *Pseudovibrio* sp. FO-BEG1 produces Fe-chelating molecules. This phenomenon was also observed in two out of three *Roseobacter* strains. A more extended screening including strains from different phylogenetic groups would be needed to understand how common is this process amongst marine bacteria. As yet, the nature of the compounds secreted by *Pseudovibrio* is unclear. Recently, D’Alvise et al. (2016) reported that TDA and an uncharacterised precursor molecule (pre-TDA) interact with Fe. *Pseudovibrio* produces TDA under P_i_ limitation (fraction 4 and Romano et al., 2014, 2015). However, in our experiments fraction 1 (**Figure 4**), which reacted in the CAS-assay and with Ga in the MS analysis, did not contain TDA, whereas the purified fraction containing TDA (fraction 4; **Figure 4A**) reacted only mildly with the CAS reagent and did not bind to Ga during the MS experiments. D’Alvise et al. (2016) pointed out that acidification triggers the rapid conversion of pre-TDA into TDA. In our study, the partial purification was always performed under acidic conditions; thus, if pre-TDA was present in the supernatant it would have been converted to TDA. Therefore, the presence of a compound analoges to pre-TDA in the active fraction 1 is unlikely. Overall, our data suggest that *Pseudovibrio* sp. FO-BEG1 produces multiple compounds, other than TDA, with chelating properties during P_i_-limited growth.

Considering the geochemical interaction between P_i_ and other Fe-minerals, such as Fe-oxyhydroxides, the increase of dissolved Fe at the end of the exponential growth phase and the secretion of Fe-chelating molecules (**Figures 2A**, **3**) led us to the hypothesis that in the environment P_i_-limited bacteria might dissolve Fe-oxyhydroxides or other Fe-containing minerals in order to access the adsorbed P_i_. This process may serve as an as yet unrecognized link between the biogeochemical cycles of P and Fe, and may be an important microbially driven process, which would allow bacteria to access an additional P_i_ pool trapped in Fe minerals. Such phenomenon might be of importance especially in environments where P_i_ availability is reduced by adsorption onto different Fe-containing minerals, or in areas where allochthonous P_i_-coated Fe-minerals are found, such as in some oxic marine sediments, regions affected by hydrothermal vent plumes, and many coastal areas (Feely et al., 1998; Paytan and McLaughlin, 2007). Additional experiments, using different Fe:P_i_ ratios, different nutrient regimes simulating various environmental conditions, and different bacterial strains and natural bacterial communities, together with the characterization of the Fe-chelating molecules produced under P_i_ limitation, will allow to verify this hypothesis, providing novel insights into the biogeochemical cycles of both Fe and P.

The ability of microorganisms to mobilize elements via dissolving minerals is a known phenomenon in soil science (Uroz et al., 2009). The general accepted idea is that the dissolution of P_i_-containing minerals is most likely due to the decrease in pH resulting from the secretion of organic acids (i.e., oxalic acid, citric acid). Thus far, there is only indirect evidence and data from abiotic experiments suggesting that the chelating properties of some organic acids, or other chelating molecules, facilitate this process by dissolving the cations (i.e., Ca, Al) of P_i_-containing minerals (Whitelaw et al., 1999; Kim et al., 2005; Osorio and Habte, 2012; Koele et al., 2014). In our experiments, the pH of the medium during bacterial growth was always ≥7 (data not shown). Moreover, the SEC, as well as the data from both the dialysis experiment, and the MS suggested that Fe was bound to organic molecules with molecular masses mostly higher than those of the organic acids secreted by P_i_ mobilizing soil bacteria, even if the stoichiometric ratio of 2:2 common for the citric acid-Fe complex is considered (551.8 Da; Shweky et al., 1994). Thus, overall, our data are consistent with the presence of multiple compounds able to chelate Fe having molecular masses comparable to those of known siderophores (i.e., enterobactin, 669.6 Da).

Except for *P. inhibens*, gene clusters responsible for the production of siderophores could not be identified in the genomes of the investigated strains, suggesting that Fe chelation is mediated by molecules different from known siderophores. Generally, bacteria produce siderophore-like molecules in response to Fe starvation, as a strategy to increase Fe uptake. In strain FO-BEG1, the EDTA-experiment along with previous proteomic data (Romano et al., 2015) showed that −P_i_-growing cells were not Fe starved and therefore that the secreted Fe-chelators had a biological function other than Fe-scavenging. While additional roles for siderophore-like compounds have been recently described (reviewed in Johnstone and Nolan, 2015), the production of these molecules in response to P_i_ limitation has been reported only in the pathogenic bacterium *Pseudomonas aeruginosa*, in a study on the effect of P_i_ limitation on virulence (Zaborin et al., 2009). To the best of our knowledge, the data we present provide the first evidence that P_i_ limitation triggers the production of Fe-chelating molecules in marine bacteria.

### Evidence for an Interlink between the Metabolism of Phosphate, Iron, and Cobalt

The increased biotic removal of Fe and Co observed under −P_i_ conditions might have derived from a more pronounced cellular uptake or from a more intense adsorption onto cellular surface due to cellular surface modifications occurring under P_i_ limitation. The absence of such an increased biotic removal in the P_i_-limited *Roseobacter* cultures suggests that either the growth conditions we used did not trigger a P_i_-limited response as pronounced as in *Pseudovibrio*, or that the process responsible for the metal decrease is strain specific and it might reflect the adaptation of *Pseudovibrio* to specific environmental niches. As yet, we cannot definitely estimate to what extent cellular adsorption and uptake contributed to the decrease of metals in the cultures. However, experiments on metal adsorption conducted on other bacteria, as well as previously proteomic and physiological data in *Pseudovibrio* suggest that cellular uptake might have played a major role in the decrease of Co and Fe in the −P_i_ cultures. For example, for many bacteria it has been shown that the intensity of the metal adsorption onto cell surface is affected by the age of the cultures, rather than the nutrient regimes used for cultivation (Borrok et al., 2004; Haas, 2004; Guiné et al., 2007; Pokrovsky et al., 2008). This suggests that the metal adsorption onto cellular surface is probably responsible for a part of the Fe and Co decrease observed in the *Pseudovibrio* cultures, but unlikely explains alone the total decrease in metal concentrations observed under P_i_-limited conditions.

In previous proteomic analyses performed comparing the expression profiles of *Pseudovibrio* cells under the two P_i_ regimes, two proteins homologous to bacterioferritin were strongly up-regulated under P_i_ limitation (PSE_1030 and PSE_3844; Romano et al., 2015). These proteins have been described to be involved in Fe storage in other bacteria, strengthening the hypothesis that *Pseudovibrio* cells growing under P_i_ limitation take up and store a larger amount of Fe than cells growing under P_i_ surplus. Luxury Fe uptake was described in phytoplankton (Kustka et al., 2003; Shire and Kustka, 2015) and it might be an important strategy to overcome period of future Fe limitation, especially in environments in which the availability of this nutrient is variable both temporally and spatially, such as in coastal waters, where *Pseudovibrio* related bacteria often occur (Shieh et al., 2004; Hosoya and Yokota, 2007).

Under conditions of P_i_ limitation, bacteria produce an array of enzymes to recover P_i_ from organic molecules. The majority of these enzymes have metal cations as cofactors, and this might explain why P_i_ limitation affects the uptake of trace elements in *Pseudovibrio* cultures. For example, most of the known forms of alkaline phosphatases (APs), an enzyme that breaks C-O-P bonds in phosphoesters, need zinc (Zn) or calcium (Ca) for activity, and it was recently discovered that PhoX, the most common AP amongst marine bacteria, requires Fe (Sebastian and Ammerman, 2009; Kathuria and Martiny, 2011; Yong et al., 2014). Co as well was found to be required in some APs and in metallozymes involved in phosphonate degradation (molecules containing C-P bonds; Simpson and Vallee, 1969; Hulett et al., 1991; Wojciechowski et al., 2002; Kulakova et al., 2003; Gong et al., 2005; Jakuba et al., 2008). In the genome of *Pseudovibrio* sp. FO-BEG1 different APs, including PhoA (PSE_2813) and PhoX (PSE_1012), and phosphonate degrading enzymes are encoded. Consistent with the P_i_ starvation response described in other bacteria, under P_i_ limitation we detect an increase in the APs activity and in the expression of enzymes involved in phosphonate metabolism in the *Pseudovibrio* cultures (PSE_3629, PSE_3630, PSE_4852, PSE_4857; Romano et al., 2015). Overall, these data suggest that under P_i_ limitation *Pseudovibrio* cells would require a higher amount of Co and Fe for the metallozymes needed to scavenge P_i_ from organic molecules, accounting, at least partially, for the more pronounced decrease of Co and Fe in the −P_i_ cultures. This conclusion is consistent with what as been previously observed for Co in freshwater phytoplankton (Ji and Sherrell, 2008), and strengthens the hypothesis that the low level of this element measured in some P_i_-limited regions, such as the Sargasso Sea, reflects the physiological response of microorganisms to P_i_ limitation.

## Conclusion

Our previous investigations on *Pseudovibrio* sp. strain FO-BEG1 demonstrated the drastic effect of P_i_ limitation on central metabolism, antibiotic production, expression of virulence-related genes, and the overall pattern of secreted metabolites (Romano et al., 2014, 2015). The changes in the metabolism of micronutrients we herein report underlines the pleiotropic effect that P_i_ limitation has on the overall cell physiology, and strongly points toward a tight interlink between the P_i_ metabolism and the metabolism of other micronutrients in marine bacteria. Our findings led us to purpose a new mechanism that bacteria might use to access P_i_ via the dissolution of Fe-minerals, such as Fe-oxyhydroxydes, which trap P_i_ in many terrestrial and aquatic environments. The extend and the environmental relevance of this phenomenon will need to be experimentally verified, and if confirmed, this process will represent an additional aspect to take into account when considering the mechanistic interconnection between the biogeochemical cycles of P_i_ and Fe. Moreover, the secretion of Fe-chelators under P_i_ limitation will open the need to a re-interpretation of the role that siderophore-like molecules have in the environment, especially considering the regulatory processes behind their production under not Fe-limiting conditions.

## Author Contributions

The work was designed by SR, HS-V, and VB. SR and VB performed the experiments. All data were analyzed by SR in collaboration with VB, HS-V, MK, and TD. SR wrote the manuscript, including comments of all co-authors. All authors reviewed and approved the final version of the manuscript.

## Conflict of Interest Statement

The authors declare that the research was conducted in the absence of any commercial or financial relationships that could be construed as a potential conflict of interest.

## Supplementary Material

FIGURE S1 **Schematic overview of the cultivation experiments performed with *Pseudovibrio* sp. strain FO-BEG1. (A)** Description of the original cultivation experiments performed using CM medium amended with an acidified trace element solution. **(B)** In the refresh experiment, cells were pre-grown under −P_i_ conditions, until the previously detected maximum Fe uptake was reached. Cells were then harvested and re-inoculated into fresh Fe-free −P_i_ CM medium. **(C)** In the EDTA-experiment, strain FO-BEG1 was cultivated in +P_i_ and −P_i_ CM medium containing an excess of EDTA, in order to complex Fe, and avoid Fe precipitation.

FIGURE S2 **Growth curve and phosphate uptake measured in the *Roseobacter* cultures.** Cell density (triangles) and phosphate concentrations (circles) over time in cultures grown under +P_i_ (empty symbols) and −P (filled symbols) conditions. **(A)** *Phaeobacter inhibens* strain DSM-17395; **(B)** *Phaeobacter inhibens* strain DSM-16374; **(C)** *Ruegeria pomeroyi* strain DSM-15171. The error bars represent the standard deviation of at least biological triplicates. Strain DSM-16374 formed tight flocs that increased the variability of the cell counting data.

## References

[B1] Aeckersberg F. Bak F. Widdel F. (1991). Anaerobic oxidation of saturated hydrocarbons to CO2 by a new type of sulfate-reducing bacterium. *Arch. Microbiol.* 156 5–14. 10.1007/BF00418180

[B2] Andrews S. C. Robinson A. K. Rodríguez-Quiñones F. (2003). Bacterial iron homeostasis. *FEMS Microbiol. Rev.* 27 215–237. 10.1016/S0168-6445(03)00055-X 12829269

[B3] Berner R. A. (1973). Phosphate removal from sea water by adsorption on volcanogenic ferric oxides. *Earth Planet. Sci. Lett.* 18 77–86. 10.1016/0012-821X(73)90037-X

[B4] Bondarev V. Richter M. Romano S. Piel J. Schwedt A. Schulz-Vogt H. N. (2013). The genus *Pseudovibrio* contains metabolically versatile bacteria adapted for symbiosis. *Environ. Microbiol.* 15 2095–2113. 10.1111/1462-2920.12123 23601235 PMC3806328

[B5] Borrok D. Fein J. B. Tischler M. O’Loughlin E. Meyer H. Liss M. (2004). The effect of acidic solutions and growth conditions on the adsorptive properties of bacterial surfaces. *Chem. Geol.* 209 107–119. 10.1016/j.chemgeo.2004.04.025

[B6] Brysch K. Schneider C. Fuchs G. Widdel F. (1987). Lithoautotrophic growth of sulfate- reducing bacteria, and description of *Desulfobacterium autotrophicum* gen. nov., sp. nov. *Arch. Microbiol.* 148 264–274. 10.1007/BF00456703

[B7] Châtellier X. Grybos M. Abdelmoula M. Kemner K. M. Leppard G. G. Mustin C. (2013). Immobilization of P by oxidation of Fe(II) ions leading to nanoparticle formation and aggregation. *Appl. Geochem.* 35 325–339. 10.1016/j.apgeochem.2013.04.019

[B8] Cosmidis J. Benzerara K. Morin G. Busigny V. Lebeau O. Jezequel D. (2014). Biomineralization of iron-phosphates in the water column of Lake Pavin (Massif Central, France). *Geochim. Cosmochim. Acta* 126 78–96. 10.1016/j.gca.2013.10.037

[B9] Cotner J. B. Ammerman J. W. Peele E. R. Bentzen E. (1997). Phosphorus-limited bacterioplankton growth in the Sargasso Sea. *Aquat. Microb. Ecol.* 13 141–149. 10.3354/ame013141

[B10] D’Alvise P. W. Phippen C. B. W. Nielsen K. F. Gram L. (2016). Influence of iron on production of the antibacterial compound tropodithietic acid and its noninhibitory analog in *Phaeobacter inhibens*. *Appl. Environ. Microbiol.* 82 502–509. 10.1128/AEM.02992-15 PMC4711134 26519388

[B11] Feely R. A. Trefry J. H. Lebon G. T. German C. R. (1998). The relationship between P/Fe and V/Fe ratios in hydrothermal precipitates and dissolved phosphate in seawater. *Geophys. Res. Lett.* 25 2253–2256. 10.1029/98GL01546

[B12] Froelich P. N. Bender M. L. Heath G. R. (1977). Phosphorus accumulation rates in metalliferous sediments on the East Pacific Rise. *Earth Planet. Sci. Lett.* 34 351–359. 10.1016/0012-821X(77)90044-9

[B13] Gledhill M. McCormack P. Ussher S. Achterberg E. P. Mantoura R. F. C. Worsfold P. J. (2004). Production of siderophore type chelates by mixed bacterioplankton populations in nutrient enriched seawater incubations. *Mar. Chem.* 88 75–83. 10.1016/j.marchem.2004.03.003

[B14] Gong N. Chen C. Xie L. Chen H. Lin X. Zhang R. (2005). Characterization of a thermostable alkaline phosphatase from a novel species *Thermus yunnanensis* sp. nov. and investigation of its cobalt activation at high temperature. *Biochim. Biophys. Acta* 1750 103–111. 10.1016/j.bbapap.2005.05.007 15955749

[B15] Guiné V. Martins J. M. F. Causse B. Durand A. Gaudet J.-P. Spadini L. (2007). Effect of cultivation and experimental conditions on the surface reactivity of the metal-resistant bacteria *Cupriavidus metallidurans* CH34 to protons, cadmium and zinc. *Chem. Geol.* 236 266–280. 10.1016/j.chemgeo.2006.10.001

[B16] Haas J. R. (2004). Effects of cultivation conditions on acid–base titration properties of *Shewanella putrefaciens*. *Chem. Geol.* 209 67–81. 10.1016/j.chemgeo.2004.04.022

[B17] Hansen H. P. Karoleff F. (1999). “Determination of nutrients,” in *Methods of Seawater Analysis*, eds Grasshoff K. Kremling K. Ehrhardt M. (Weinheim: Wiley-VCH), 159–226. 10.1002/9783527613984.ch10

[B18] Hosoya S. Yokota A. (2007). *Pseudovibrio japonicus* sp. nov., isolated from coastal seawater in Japan. *Int. J. Syst. Evol. Microbiol.* 57 1952–1955. 10.1099/ijs.0.64922-0 17766853

[B19] Hsieh Y.-J. Wanner B. L. (2010). Global regulation by the seven-component Pi signaling system. *Curr. Opin. Microbiol.* 13 198–203. 10.1016/j.mib.2010.01.014 20171928 PMC2847643

[B20] Hulett F. M. Kim E. E. Bookstein C. Kapp N. V. Edwards C. W. Wyckoff H. W. (1991). *Bacillus subtilis* alkaline phosphatases III and IV. Cloning, sequencing, and comparisons of deduced amino acid sequence with *Escherichia coli* alkaline phosphatase three-dimensional structure. *J. Biol. Chem.* 266 1077–1084. 1898729

[B21] Ishige T. Krause M. Bott M. Wendisch V. F. Sahm H. (2003). The phosphate starvation stimulon of *Corynebacterium glutamicum* determined by DNA microarray snalyses. *J. Bacteriol.* 185 4519–4529. 10.1128/JB.185.15.4519-4529.2003 12867461 PMC165763

[B22] Jakuba R. W. Moffett J. W. Dyhrman S. T. (2008). Evidence for the linked biogeochemical cycling of zinc, cobalt, and phosphorus in the western North Atlantic Ocean. *Glob. Biogeochem. Cycles* 22 GB4012 10.1029/2007GB003119

[B23] Ji Y. Sherrell R. M. (2008). Differential effects of phosphorus limitation on cellular metals in *Chlorella* and *Microcystis*. *Limnol. Oceanogr.* 53 1790–1804. 10.4319/lo.2008.53.5.1790

[B24] Johnstone T. Nolan E. M. (2015). Beyond iron: non-classical biological functions of bacterial siderophores. *Dalton Trans.* 44 6320–6339. 10.1039/C4DT03559C 25764171 PMC4375017

[B25] Kathuria S. Martiny A. C. (2011). Prevalence of a calcium-based alkaline phosphatase associated with the marine cyanobacterium *Prochlorococcus* and other ocean bacteria. *Environ. Microbiol.* 13 74–83. 10.1111/j.1462-2920.2010.02310.x 20649645

[B26] Kim Y. Bae B. Choung Y. (2005). Optimization of biological phosphorus removal from contaminated sediments with phosphate-solubilizing microorganisms. *J. Biosci. Bioeng.* 99 23–29. 10.1263/jbb.99.23 16233749

[B27] Koele N. Dickie I. A. Blum J. D. Gleason J. D. de Graaf L. (2014). Ecological significance of mineral weathering in ectomycorrhizal and arbuscular mycorrhizal ecosystems from a field-based comparison. *Soil Biol. Biochem.* 69 63–70. 10.1016/j.soilbio.2013.10.041

[B28] Krol E. Becker A. (2004). Global transcriptional analysis of the phosphate starvation response in *Sinorhizobium meliloti* strains 1021 and 2011. *Mol. Genet. Genomics* 272 1–17. 10.1007/s00438-004-1030-8 15221452

[B29] Kulakova A. N. Wisdom G. B. Kulakov L. A. Quinn J. P. (2003). The purification and characterization of phosphonopyruvate hydrolase, a novel carbon-phosphorus bond cleavage enzyme from *Variovorax* sp. Pal2. *J. Biol. Chem.* 278 23426–23431. 10.1074/jbc.M301871200 12697754

[B30] Kustka A. B. Sañudo-Wilhelmy S. A. Carpenter E. J. Capone D. Burns J. Sunda W. G. (2003). Iron requirements for dinitrogen- and ammonium-supported growth in cultures of *Trichodesmium* (IMS 101): comparison with nitrogen fixation rates and iron:carbon ratios of field populations. *Limnol. Oceanogr.* 48 1869–1884. 10.4319/lo.2003.48.5.1869

[B31] Martín J. F. (2004). Phosphate control of the biosynthesis of antibiotics and other secondary metabolites is mediated by the PhoR-PhoP system: an unfinished story. *J. Bacteriol.* 186 5197–5201. 10.1128/JB.186.16.5197-5201.2004 15292120 PMC490900

[B32] Millero F. J. (1998). Solubility of Fe(III) in seawater. *Earth Planet. Sci. Lett.* 154 323–329. 10.1016/S0012-821X(97)00179-9

[B33] Morrow J. R. Buttrey L. A. Berback K. A. (1992). Transesterification of a phosphate diester by divalent and trivalent metal ions. *Inorg. Chem.* 31 16–20. 10.1021/ic00027a005

[B34] Osorio N. W. Habte M. (2012). Phosphate desorption from the surface of soil mineral particles by a phosphate-solubilizing fungus. *Biol. Fertil. Soils* 49 481–486. 10.1007/s00374-012-0763-5

[B35] Paytan A. McLaughlin K. (2007). The oceanic phosphorus cycle. *Chem. Rev.* 107 563–576. 10.1021/cr0503613 17256993

[B36] Pokrovsky O. S. Martinez R. E. Golubev S. V. Kompantseva E. I. Shirokova L. S. (2008). Adsorption of metals and protons on *Gloeocapsa* sp. cyanobacteria: a surface speciation approach. *Appl. Geochem.* 23 2574–2588. 10.1016/j.apgeochem.2008.05.007

[B37] Rodríguez-García A. Barreiro C. Santos-Beneit F. Sola-Landa A. Martín J. F. (2007). Genome-wide transcriptomic and proteomic analysis of the primary response to phosphate limitation in *Streptomyces coelicolor* M145 and in a ΔphoP mutant. *Proteomics* 7 2410–2429. 10.1002/pmic.200600883 17623301

[B38] Romano S. Dittmar T. Bondarev V. Weber R. J. M. Viant M. R. Schulz-Vogt H. N. (2014). Exo-metabolome of *Pseudovibrio* sp. FO-BEG1 analyzed by ultra-high resolution mass spectrometry and the effect of phosphate limitation. *PLoS ONE* 9:e96038 10.1371/journal.pone.0096038 PMC4008564 24787987

[B39] Romano S. Fernàndez-Guerra A. Reen F. J. Glöckner F. O. Crowley S. P. O’Sullivan O. (2016). Comparative genomic analysis reveals a diverse repertoire of genes involved in Prokaryote-Eukaryote interactions within the *Pseudovibrio* Genus. *Front. Microbiol.* 7:387 10.3389/fmicb.2016.00387 PMC4811931 27065959

[B40] Romano S. Schulz-Vogt H. N. González J. M. Bondarev V. (2015). Phosphate limitation induces drastic physiological changes, virulence-related gene expression, and secondary metabolite oroduction in *Pseudovibrio* sp. Strain FO-BEG1. *Appl. Environ. Microbiol.* 81 3518–3528. 10.1128/AEM.04167-14 25769826 PMC4407226

[B41] Saito M. A. Moffett J. W. (2002). Temporal and spatial variability of cobalt in the Atlantic Ocean. *Geochim. Cosmochim. Acta* 66 1943–1953. 10.1016/S0016-7037(02)00829-3

[B42] Schwedt A. Seidel M. Dittmar T. Simon M. Bondarev V. Romano S. (2015). Substrate use of *Pseudovibrio* sp. growing in ultra-oligotrophic seawater. *PLOS ONE* 10:e0121675 10.1371/journal.pone.0121675 PMC4380363 25826215

[B43] Schwyn B. Neilands J. B. (1987). Universal chemical assay for the detection and determination of siderophores. *Anal. Biochem.* 160 47–56. 10.1016/0003-2697(87)90612-9 2952030

[B44] Sebastian M. Ammerman J. W. (2009). The alkaline phosphatase PhoX is more widely distributed in marine bacteria than the classical PhoA. *ISME J.* 3 563–572. 10.1038/ismej.2009.10 19212430

[B45] Seemann T. (2014). Prokka: rapid prokaryotic genome annotation. *Bioinformatics* 30 2068–2069. 10.1093/bioinformatics/btu153 24642063

[B46] Shieh W. Y. Lin Y.-T. Jean W. D. (2004). *Pseudovibrio denitrificans* gen. nov., sp. nov., a marine, facultatively anaerobic, fermentative bacterium capable of denitrification. *Int. J. Syst. Evol. Microbiol.* 54 2307–2312. 10.1099/ijs.0.63107-0 15545476

[B47] Shire D. M. Kustka A. B. (2015). Luxury uptake, iron storage and ferritin abundance in *Prochlorococcus marinus* (Synechococcales) strain MED4. *Phycologia* 54 398–406. 10.2216/14-109.1

[B48] Shweky I. Bino A. Goldberg D. P. Lippard S. J. (1994). Syntheses, structures, and magnetic properties of two dinuclear Iron(III) citrate complexes. *Inorg. Chem.* 33 5161–5162. 10.1021/ic00101a001

[B49] Simpson R. T. Vallee B. L. (1969). Zinc and cobalt alkaline phosphatases. *Ann. N. Y. Acad. Sci.* 166 670–695. 10.1111/j.1749-6632.1969.tb54308.x 4907876

[B50] Thingstad T. F. Krom M. D. Mantoura R. F. C. Flaten G. A. F. Groom S. Herut B. (2005). Nature of phosphorus limitation in the ultraoligotrophic eastern Mediterranean. *Science* 309 1068–1071. 10.1126/science.1112632 16099984

[B51] Uroz S. Calvaruso C. Turpault M.-P. Frey-Klett P. (2009). Mineral weathering by bacteria: ecology, actors and mechanisms. *Trends Microbiol.* 17 378–387. 10.1016/j.tim.2009.05.004 19660952

[B52] Wandersman C. Delepelaire P. (2004). Bacterial iron sources: from siderophores to hemophores. *Annu. Rev. Microbiol.* 58 611–647. 10.1146/annurev.micro.58.030603.123811 15487950

[B53] Weber T. Blin K. Duddela S. Krug D. Kim H. U. Bruccoleri R. (2015). antiSMASH 3.0—a comprehensive resource for the genome mining of biosynthetic gene clusters. *Nucleic Acids Res.* 43 W237–W243. 10.1093/nar/gkv437 25948579 PMC4489286

[B54] Whitelaw M. A. Harden T. J. Helyar K. R. (1999). Phosphate solubilisation in solution culture by the soil fungus *Penicillium radicum*. *Soil Biol. Biochem.* 31 655–665. 10.1016/S0038-0717(98)00130-8

[B55] Widdel F. Pfennig N. (1984). “Dissimilatory sulfate- or sulfur-reducing bacteria,” in *Bergey’s Manual of Systematic Bacteriology*, eds Krieg N. R. Holt J. C. (Baltimore, MD: Williams & Wilkins).

[B56] Wojciechowski C. L. Cardia J. P. Kantrowitz E. R. (2002). Alkaline phosphatase from the hyperthermophilic bacterium *T. maritima* requires cobalt for activity. *Protein Sci.* 11 903–911. 10.1110/ps.4260102 11910033 PMC2373536

[B57] Wu J. Sunda W. Boyle E. A. Karl D. M. (2000). Phosphate depletion in the western north Atlantic ocean. *Science* 289 759–762. 10.1126/science.289.5480.759 10926534

[B58] Yong S. C. Roversi P. Lillington J. Rodriguez F. Krehenbrink M. Zeldin O. B. (2014). A complex iron-calcium cofactor catalyzing phosphotransfer chemistry. *Science* 345 1170–1173. 10.1126/science.1254237 25190793 PMC4175392

[B59] Zaborin A. Romanowski K. Gerdes S. Holbrook C. Lepine F. Long J. (2009). Red death in *Caenorhabditis elegans* caused by *Pseudomonas aeruginosa* PAO1. *Proc. Natl. Acad. Sci. U.S.A.* 106 6327–6332. 10.1073/pnas.0813199106 19369215 PMC2669342

