# Supplementary material for: Phosphate Limitation Triggers the Dissolution of Precipitated Iron by the Marine Bacterium Pseudovibrio sp. FO-BEG1
Source: Front Microbiol. 2017 Mar 14;8:364. doi: 10.3389/fmicb.2017.00364 (PMC5348524; doi:10.3389/fmicb.2017.00364)
Supplement: FIGURE S1 — Schematic overview of the cultivation experiments performed with Pseudovibrio sp. strain FO-BEG1. (A) Description of the original cultivation experiments performed using CM medium amended with an acidified trace element solution. (B) In the refresh experiment, cells were pre-grown under −Pi conditions, until the previously detected maximum Fe uptake was reached. Cells were then harvested and re-inoculated into fresh Fe-free −Pi CM medium. (C) In the EDTA-experiment, strain FO-BEG1 was cultivated in +Pi and −Pi CM medium containing an excess of EDTA, in order to complex Fe, and avoid Fe precipitation. [file Image_1.pdf]

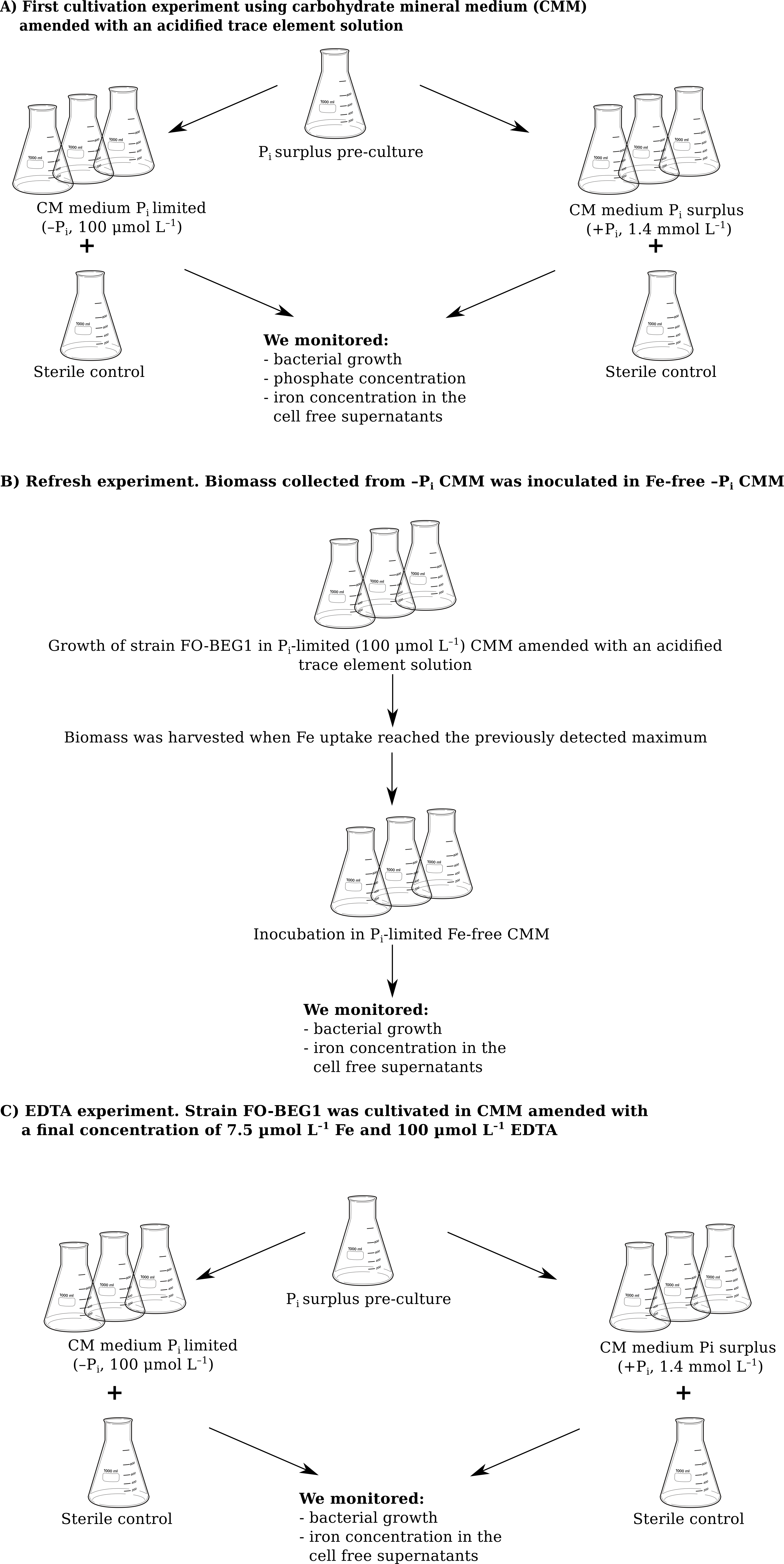
A) First cultivation experiment using carbohydrate mineral medium (CMM) amended with an acidified trace element solution
P_i surplus pre-culture
CM medium P_i limited (–P_i, 100 µmol L^{-1})
+
CM medium P_i surplus (+P_i, 1.4 mmol L^{-1})
+
Sterile control
We monitored:
- bacterial growth
- phosphate concentration
- iron concentration in the cell free supernatants
Sterile control
1000 ml
B) Refresh experiment. Biomass collected from –P_i CMM was inoculated in Fe-free –P_i CMM
Growth of strain FO-BEG1 in P_i-limited (100 µmol L^{-1}) CMM amended with an acidified trace element solution
Biomass was harvested when Fe uptake reached the previously detected maximum
Inocubation in P_i-limited Fe-free CMM
We monitored:
- bacterial growth
- iron concentration in the cell free supernatants
C) EDTA experiment. Strain FO-BEG1 was cultivated in CMM amended with a final concentration of 7.5 µmol L^{-1} Fe and 100 µmol L^{-1} EDTA
P_i surplus pre-culture
CM medium P_i limited (–P_i, 100 µmol L^{-1})
+
CM medium Pi surplus (+P_i, 1.4 mmol L^{-1})
+
Sterile control
We monitored:
- bacterial growth
- iron concentration in the cell free supernatants
Sterile control
