# Supplementary figures and images for: Phosphate Limitation Triggers the Dissolution of Precipitated Iron by the Marine Bacterium Pseudovibrio sp. FO-BEG1
Source: Front Microbiol. 2017 Mar 14;8:364. doi: 10.3389/fmicb.2017.00364 (PMC5348524; doi:10.3389/fmicb.2017.00364)

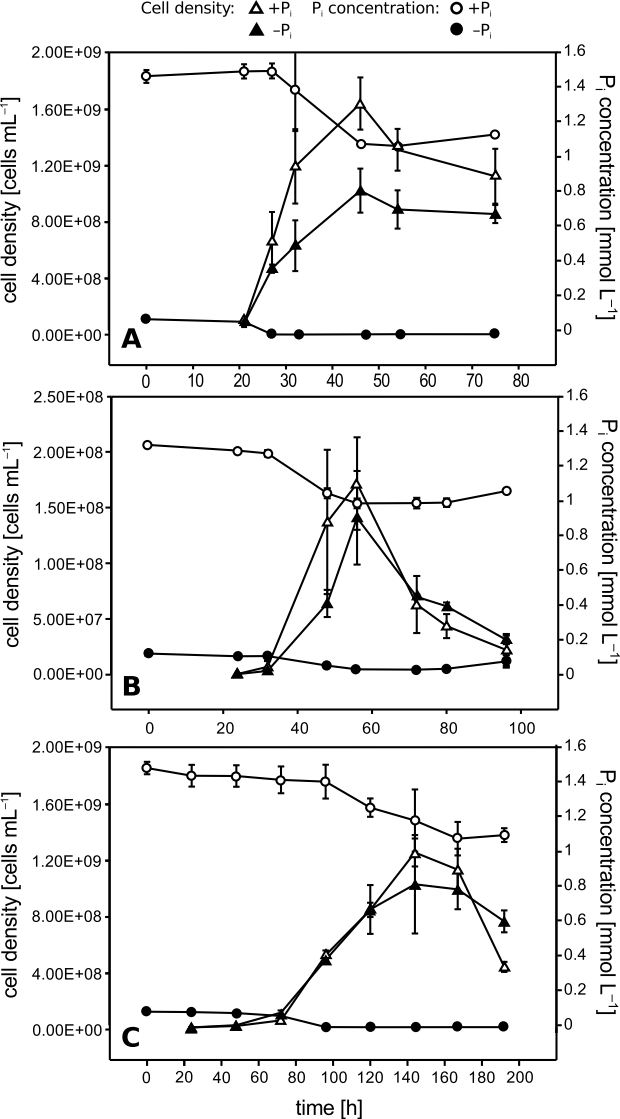

Supplement: FIGURE S2 — Growth curve and phosphate uptake measured in the Roseobacter cultures. Cell density (triangles) and phosphate concentrations (circles) over time in cultures grown under +Pi (empty symbols) and −P (filled symbols) conditions. (A) Phaeobacter inhibens strain DSM-17395; (B) Phaeobacter inhibens strain DSM-16374; (C) Ruegeria pomeroyi strain DSM-15171. The error bars represent the standard deviation of at least biological triplicates. Strain DSM-16374 formed tight flocs that increased the variability of the cell counting data. [file Image_2.PDF]
